# An Automated Method for Classifying Liver Lesions in Contrast-Enhanced Ultrasound Imaging Based on Deep Learning Algorithms

**DOI:** 10.3390/diagnostics13061062

**Published:** 2023-03-10

**Authors:** Mădălin Mămuleanu, Cristiana Marinela Urhuț, Larisa Daniela Săndulescu, Constantin Kamal, Ana-Maria Pătrașcu, Alin Gabriel Ionescu, Mircea-Sebastian Șerbănescu, Costin Teodor Streba

**Affiliations:** 1 Department of Automatic Control and Electronics, University of Craiova, 200585 Craiova, Romania; 2 Oncometrics S.R.L., 200677 Craiova, Romania; 3 Department of Gastroenterology, Emergency County Hospital of Craiova, 200642 Craiova, Romania; 4 Department of Gastroenterology, Research Center of Gastroenterology and Hepatology, University of Medicine and Pharmacy of Craiova, 200349 Craiova, Romania; 5 Department of Pulmonology, University of Medicine and Pharmacy of Craiova, 200349 Craiova, Romania; 6 Department of Hematology, University of Medicine and Pharmacy of Craiova, 200349 Craiova, Romania; 7 Department of History of Medicine, University of Medicine and Pharmacy of Craiova, 200349 Craiova, Romania; 8 Department of Medical Informatics and Statistics, University of Medicine and Pharmacy of Craiova, 200349 Craiova, Romania

**Keywords:** contrast-enhanced ultrasound, liver, hepatocarcinoma, image segmentation, deep learning

## Abstract

Background: Contrast-enhanced ultrasound (CEUS) is an important imaging modality in the diagnosis of liver tumors. By using contrast agent, a more detailed image is obtained. Time-intensity curves (TIC) can be extracted using a specialized software, and then the signal can be analyzed for further investigations. Methods: The purpose of the study was to build an automated method for extracting TICs and classifying liver lesions in CEUS liver investigations. The cohort contained 50 anonymized video investigations from 49 patients. Besides the CEUS investigations, clinical data from the patients were provided. A method comprising three modules was proposed. The first module, a lesion segmentation deep learning (DL) model, handled the prediction of masks frame-by-frame (region of interest). The second module performed dilation on the mask, and after applying colormap to the image, it extracted the TIC and the parameters from the TIC (area under the curve, time to peak, mean transit time, and maximum intensity). The third module, a feed-forward neural network, predicted the final diagnosis. It was trained on the TIC parameters extracted by the second model, together with other data: gender, age, hepatitis history, and cirrhosis history. Results: For the feed-forward classifier, five classes were chosen: hepatocarcinoma, metastasis, other malignant lesions, hemangioma, and other benign lesions. Being a multiclass classifier, appropriate performance metrics were observed: categorical accuracy, F1 micro, F1 macro, and Matthews correlation coefficient. The results showed that due to class imbalance, in some cases, the classifier was not able to predict with high accuracy a specific lesion from the minority classes. However, on the majority classes, the classifier can predict the lesion type with high accuracy. Conclusions: The main goal of the study was to develop an automated method of classifying liver lesions in CEUS video investigations. Being modular, the system can be a useful tool for gastroenterologists or medical students: either as a second opinion system or a tool to automatically extract TICs.

## 1. Introduction

In recent years, with the evolution of technology and algorithms, artificial intelligence has had a significant impact in the healthcare field. Machine learning or deep learning algorithms are used to either predict the malignancy of a lesion or to segment a lesion in a medical imaging [1,2,3,4]. Ultrasound liver investigation is a non-invasive method with relatively low cost, and it can be effective in evaluating multiple types of liver lesions.

Contrast-enhanced ultrasound (CEUS) has become an increasingly important imaging modality in the diagnosis and management of liver tumors. CEUS combines the use of a contrast agent that is administered intravenously with ultrasound imaging to create a more detailed image of the liver [5]. Second-generation contrasting agents use stabilized microbubbles that oscillate under the compressive effect of an ultrasound (US) beam and create a non-linear response, thus providing vascular contrast. This technique has been shown to be highly accurate in diagnosing and staging liver tumors, which have different vascularity compared to normal parenchyma, allowing it to become a part of the standard of care in many medical facilities [5]. The type of pattern within a lesion reflects in the maximum contrast uptake values within the time needed for the agent to clear the surveilled area. Thus, a time-intensity curve (TIC) can be generated by a computerized system, and comparing the measurements from within the tumor and from the surrounding parenchyma may provide a quantification usable in diagnosis, staging, and prognosis [6].

The use of CEUS for the diagnosis of liver tumors has been supported by recent medical guidelines and the literature. Both the American Association for the Study of Liver Diseases (AASLD) [7] and the European Association for the Study of the Liver (EASL) [8] recommend the use of CEUS for the evaluation of focal liver lesions. The low cost of operation, large availability, and virtual lack of side effects or contraindications made CEUS a valid choice of imaging when compared to other techniques, such as computer tomography (CT) and magnetic resonance imaging (MRI) in detecting early-stage liver tumors.

However, a steep learning curve combined with an uneven training curricula and lack of consistency in the operation of the US machine can decrease the diagnostic yield of the procedure. The development and implementation of an artificial intelligence (AI)-based system that can aid the clinician in diagnosing liver malignancies through US and CEUS can thus be considered a possible solution.

## 2. Materials and Methods

The anonymized dataset used in the study was provided by the University of Medicine and Pharmacy Craiova, and it included 50 video files from CEUS liver investigations performed on the same Hitachi Aloka Arieta V70 350 (Hitachi Medical Corp.; Tokyo, Japan) with the convex C 251 probe and using SonoVue (Bracco SpA, Milan, Italy) as intravascular contrast agent. The total number of patients was 49, with 59 liver lesions. For every patient involved in the study, besides the CEUS video investigation, detailed medical results and history were provided. The anonymized video files were used to build a dataset for training a U-Net image segmentation model—basically, each frame consisted of a side-by-side representation of the B-mode US and the CEUS windows. Frames from each video investigation were automatically extracted and cropped. The mask for each frame extracted was performed manually by a senior gastroenterologist (L.D.S.) using QuPath [9]. The labels for the dataset (masks) were automatically exported from QuPath. A more detailed presentation of this method was conducted in our previous work [10]. The demographic information of the patients is presented in Table 1.

Data were extracted from a cohort of patients with either liver cirrhosis or chronic hepatitis of any etiology that had at least one focal liver lesion identifiable by US. All patients underwent a complete CEUS investigation. Other inclusion criteria were age over 18, irrespective of gender or other comorbidities, and confirmation of a liver tumor by other imaging (contrast-enhanced CT, MRI or a combination of the two), liver biopsy or surgical confirmation depending on each case, with a follow-up of at least 12 months. Patients that had missing medical records or incomplete CEUS were excluded. For every patient involved in the study, besides the CEUS video investigation, detailed medical results and history were provided.

## 3. Proposed Method

**Figure 1:**
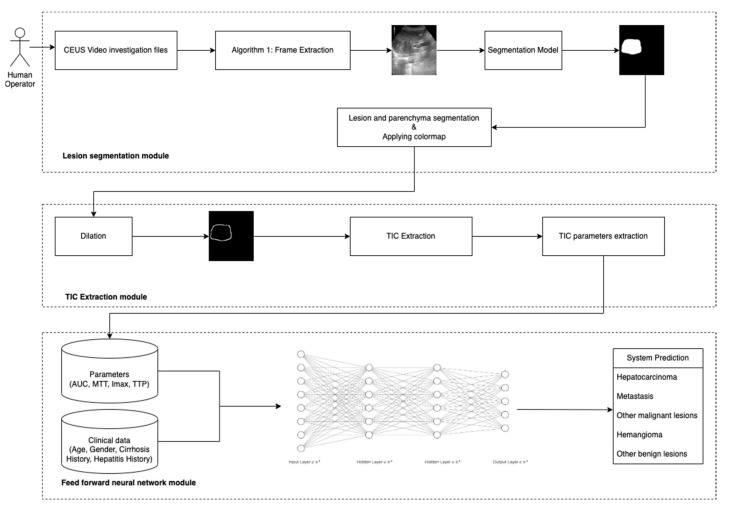
Architecture of the proposed system.

The current study presents a system based on artificial intelligence algorithms for liver lesions detection and classification in CEUS investigations. The proposed system includes three modules. The first module is an image segmentation deep learning (DL) model based on a U-Net [11] model trained on an in-house dataset. The second module represents a frame processing algorithm and a time-intensity curve (TIC) extraction algorithm. The last component, a classifier based on feed-forward neural networks, requires as input the data extracted from the TICs and clinical data of the patient. The architecture of the proposed method is presented in Figure 1.

**Figure 2:**
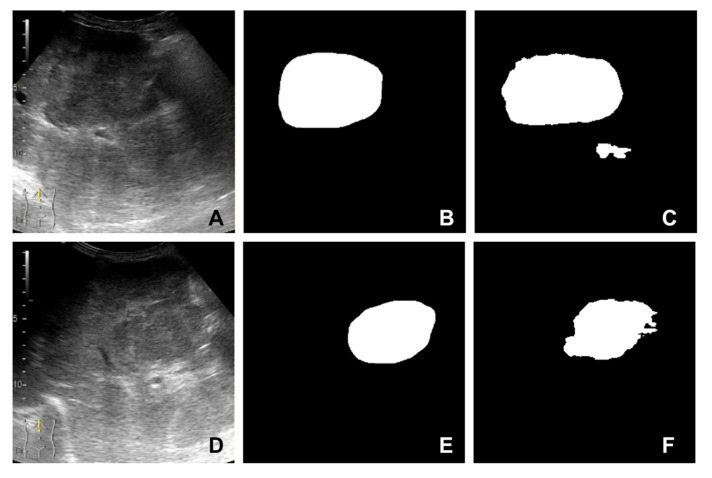
Samples extracted from the image segmentation dataset: (**A**,**D**) B-mode frame cropped from the video investigation; (**B**,**E**) mask (label) created by the senior gastroenterologist; (**C**,**F**) the mask predicted by the segmentation model.

The U-Net [11] image segmentation model obtained from our previous work [10] was used to obtain region of interest for each frame in the video investigation file. By passing a frame from the ultrasound video investigation through the presented image segmentation model, a mask of 256 by 256 pixels is obtained. This mask can be further used to isolate the lesion from parenchyma in that specific video frame. Running a video investigation through the image segmentation model, the lesion is automatically isolated from the parenchyma, frame-by-frame, in the entire file; therefore, a time-intensity curve can be extracted from the investigation. A sample of inputs and outputs of the U-Net segmentation model are presented in Figure 2A–F.

**Figure 3:**
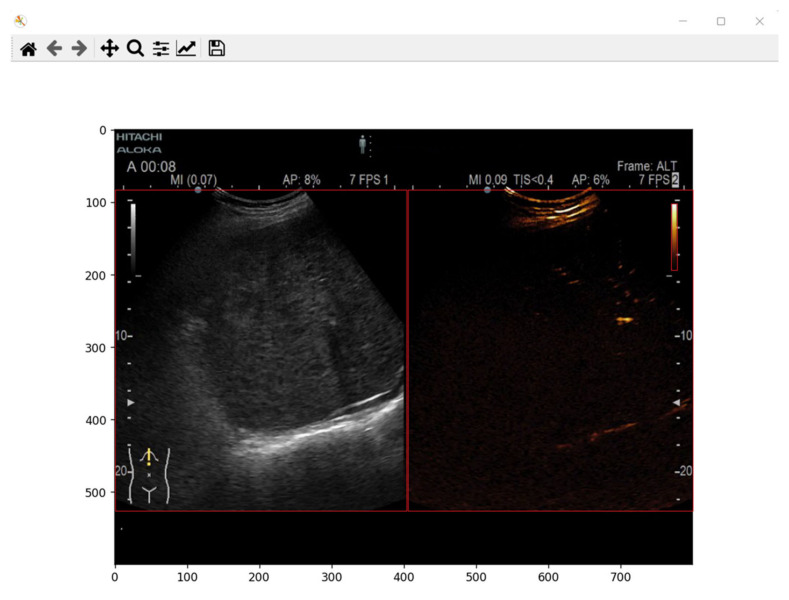
B-mode, Contrast mode, and color scale coordinates determination from one frame. Each part is marked by a rectangle with red edges.

To create the dataset and for extracting the lesion from each frame in the video investigation, boundaries were defined for each region in the video investigation. The boundaries for B-mode image were defined in our previous work [10] and were used to create the dataset but also for predicting the mask. For the contrast image section in the video investigation file, the boundaries were determined experimentally as for the B-mode image. A frame was plotted from a video investigation file, and the minimum and maximum x and y coordinates were defined. The determined values are presented in Table 2. A sample frame with the determined coordinates is presented in Figure 3.

As mentioned earlier, the goal of the present study was to create a system to automatically extract time-intensity curves and predict the malignancy of a lesion from contrast-enhanced ultrasound imaging. Therefore, besides cropping the B-mode and contrast mode images from the video investigation, the color scale was also needed. For each ultrasound device, the color scale is present in the video investigation file. To crop and obtain the color scale, the same principle was applied as for B-mode imaging and contrast mode imaging: experimentally determine the minimum and maximum x and y coordinates. The determined values for the color scale are presented in Table 3. In addition, a sample frame with the determined coordinates is presented in Figure 3.

With all the values for each section in the video investigation file determined and with the predicted mask from the image segmentation model, the lesion in B-mode or contrast mode could be extracted effortlessly. Extracting the time-intensity curves in liver contrast-enhanced ultrasound imaging is not a simple task due to multiple perturbations: patient breathing or operator moving the probe. While the lesion represented the mask predicted by the trained U-Net segmentation model, the parenchyma could not be represented by the remainder of the image (after removing the lesion). Therefore, a small region around the predicted mask had to be chosen. As the goal of the study is to present an automatically TIC extraction and prediction system, an algorithm had to be used for parenchyma determination. Dilation, which is a morphological operation, was applied on the predicted mask. Dilation is a mathematical morphology operation performed on images in which a kernel is applied to the image to obtain an output (a new image). In most of the cases, the dilation operation is applied on a binary image. Assuming that A represents the mask predicted by the U-Net segmentation model and *K* the kernel (structuring element), dilation is given by Equation (1). *A_k_* represented the translation of *A* by *k*. In our study, the size of *K*, the kernel, was 10 pixels by 10 pixels.

(1) $$A\;⊕K=\cup_{k\;\in K}A_{k}$$

**Figure 4:**
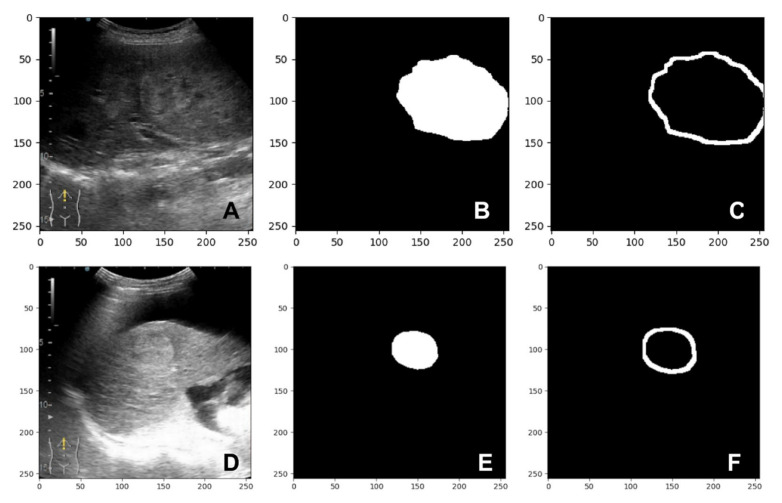
Sample frame from a patient with hepatocarcinoma: (**A**,**D**) B-mode frame; (**B**,**E**) predicted mask; (**C**,**F**) parenchyma mask.

After applying dilation on the predicted mask, a new image was obtained: the dilated mask. By applying dilation to the predicted mask pixels, the boundaries of the lesion are increased. Since both images, the predicted mask and the dilated mask, are binary images, a difference between the dilated mask and the predicted mask can be calculated to obtain a mask which represented the parenchyma surrounding the lesion. Two samples are shown in Figure 4. We present the B-mode images of the lesions in Figure 4A,D. In Figure 4B,E the predicted masks by the segmentation model for 4A and 4D are presented, and 4C presents the mask for the parenchyma surrounding the lesions.

By knowing the coordinates for each image type, B-mode or contrast mode, in the video investigation file, the mask obtained for the B-mode was applied for the contrast mode by using the proper coordinates (defined in Table 1). In addition, by knowing the coordinates of B-mode and contrast mode images, the position in the image of the B-mode and the contrast mode have been determined (left or right side). By applying the two masks obtained earlier to the contrast mode image, two images were obtained: the first image represented the lesion, isolated from the rest of the liver tissue, while the second image represented the surrounding parenchyma. To apply the colormap provided by the ultrasound device manufacturer, a custom lookup table was applied for both images. The values for the lookup table are presented in Table 1 (color map). A lookup table represents an array of data used for mapping input values to output values, and it is mostly used for functions which require a long time to compute. In our study, the lookup table was used for applying the color map to the images but also to improve the overall execution time. For each type (lesion and parenchyma), the intensity was obtained by performing the mean of the pixels in the cropped images after applying the color map. Two sets of values were obtained: the intensity for the parenchyma and the intensity for the detected lesion. The extraction of the intensity values for each type (parenchyma and lesion) was performed with a rate of one per second to give the system enough time to extract the TIC values. In CEUS, 10 frames per video investigation can be enough to extract a reliable TIC [12].

In some cases in the video investigations, the probe was lifted by the operator, and therefore computing the intensity value in this case was difficult. To treat this situation properly, an intensity value was considered valid if the difference between the current value and previous was under 75% from the previous value. If the difference was greater than 75%, the next frame was analyzed. This value was determined experimentally by analyzing the variations of the intensity in the entire frame in each second of the video file. Extracting intensity values with a frequency of one second and checking the values for possible probe lifting by the operator resulted in a curve with less noise on which parameters could be extracted. Before extracting the parameters, the TIC values were filtered using a Savitzky–Golay filter [13] with 51 window sizes and a polynomial order of 3.

The two sets of values extracted from the video investigations (intensity for the parenchyma and intensity for the lesions) were saved into comma separated values (csv) files. After saving the values into csv files, the following values were extracted from the TIC: maximum intensity, time to peak (TTP), area under the curve (AUC), and mean transit time (MTT). The entire flow described earlier has two purposes. The main purpose was to extract all the values to build a custom dataset. The second purpose was to be able to reuse parts of the method to predict the lesion type based on the video investigation file and patient data. The dataset fields are presented in Table 4, and the labels are presented in Table 5. This dataset was then used to train a feed-forward neural network model.

**Figure 5:**
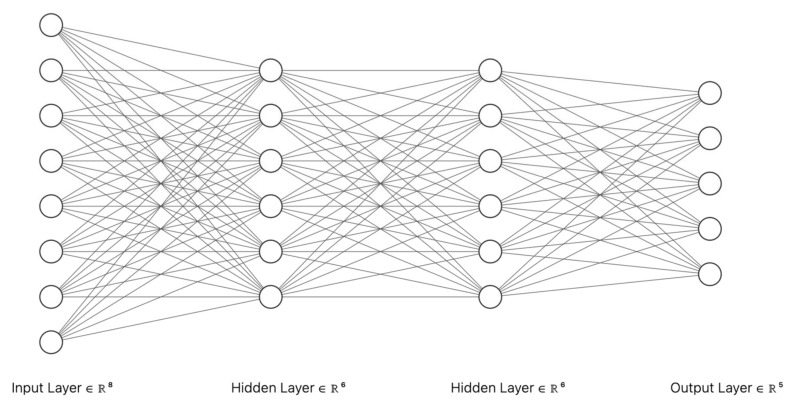
Architecture of the proposed neural network.

The proposed neural network architecture was a feed-forward neural network with eight neurons as input layer, two hidden layers with six neurons each, and 5 neurons as the output layer (one neuron for each output class presented in Table 5). The architecture of the proposed classifier is presented in Figure 5. When choosing the architecture of the neural network model, both the inference time and performance of the model were considered. The activation functions used were rectified linear unit (ReLu) for all the layer except for the output layer. For this layer, a Softmax activation function was used. The dataset created was imbalanced (40.67% of the cases were diagnosed with hepatocarcinoma). To deal with class imbalance, oversampling techniques were applied. Therefore, random samples from each class in the dataset were duplicated. This technique has been proven to be effective when dealing with class imbalance [14,15]. The dataset was split in 70% samples for training and 30% samples for validation.

The loss function used in our proposed feed-forward classifier was focal cross-entropy (FCE) proposed by Tsung-Yi Lin et al. [16]. Typically, in a multiclass classification problem, the cross-entropy loss function (Equation (2)) is used. Being a logarithmic loss function, cross-entropy introduces a large penalty for errors close to 1 and smaller penalties when the error is closer to 0. However, the cross-entropy loss function penalized all the classes equally, including the imbalanced classes.

(2) $$CE=-\sum_{i=1}^{N}t_{i}\log\left(p_{i}\right)$$

where *t_i_* is the expected label, *p_i_* represents the probability (Softmax output function) for *i*th class, and *N* is the total number of classes. Focal cross-entropy reduced the loss for well-classified samples and increased the loss for samples defined as “hard-to-classify” [16]. Focal cross-entropy was used in object detection tasks in which the class imbalance introduced a very difficult problem. Objects in the background were hard to detect if the object detection model was trained using cross-entropy [16]. FCE is given by Equation (3).

(3) $$FCE=-\sum_{i=1}^{N}\alpha_{i}{\left(i-p_{i}\right)}^{\gamma }\log\left(p_{i}\right)$$

where *N* represents the total number of classes, *p_i_* represents the probability for the *i*th class, *α* is the weighting factor, and *γ* represents the focusing parameter. The values for the weighting factor and the focusing parameter were chosen as 0.25 and 2.0, respectively. The proposed feed-forward model was trained for 100 epochs with a batch size of 50, and a learning rate of 0.0001 with the optimizer RMSProp [17]. The training was performed in the cloud, using Google Colab [18] without a graphical processing unit (GPU) and with Tensorflow version 2.9.2 [19] and Python version 3.8. The segmentation model was trained on Nvidia 3050Ti GPU with Tensorflow version 2.7.0 [19]. The feed-forward neural network from the current study was a multi-class classifier. Therefore, the performance metrics applied to binary classifiers could not be used to correctly evaluate this model. For the evaluation of the model, categorical accuracy, F1 macro, F1 micro, and Matthews correlation coefficient (MCC) were used. Categorical accuracy is defined as the number of correct predictions divided by the total number of predictions. If n represents the total number of correct predictions and N the total number of predictions, categorical accuracy (*CA*) is defined by Equation (4).

(4) $$CA=\frac{n}{N}$$

F1 micro and F1 macro scores are defined as the harmonic mean between precision micro and recall micro, respectively, precision macro and recall macro. Assuming the following notations: *TP*—true positives, *FP*—false positives, *FN*—false negatives, and *N*—the total number of classes, F1 micro and F1 macro are given by Equations (5)–(10).

(5) $$Prc_{\mu }=\frac{\sum_{i=1}^{N}TP_{i}}{\sum_{i=1}^{N}\left(TP_{i}+FP_{i}\right)}$$

(6) $$Prc_{Macro}=\frac{\sum_{i=1}^{N}\frac{TP_{i}}{TP_{i}+FP_{i}}}{N}$$

(7) $$Recall_{\mu }=\frac{\sum_{i=1}^{N}TP_{i}}{\sum_{i=1}^{N}\left(TP_{i}+FN_{i}\right)}$$

(8) $$Recall_{Macro}=\frac{\sum_{i=1}^{N}\frac{TP_{i}}{TP_{i}+FN_{i}}}{N}$$

(9) $$F1_{\mu }=2\;\frac{Prc_{\mu }\cdot \;Recall_{\mu }}{Prc_{\mu }+Recall_{\mu }}$$

(10) $$F1_{Macro}=2\;\frac{Prc_{Macro}\cdot \;Recall_{Macro}}{Prc_{Macro}+Recall_{Macro}}$$

Matthews correlation coefficient for N classes is given by Equation (11) in which N represents the total number of classes, *c* represents the total number of samples predicted correctly, *S* is the total number of samples in the batch, *t_i_* represents the number of appearances for class I, and *p_i_* is the number of predictions for class *i*.

(11) $$MCC=\frac{c\;\cdot S-\sum_{i=1}^{N}p_{i}\cdot t_{i}}{\sqrt{\left(S^{2}-\sum_{i=1}^{N}p_{i}^{2}\right)\cdot \left(s^{2}-\sum_{i=1}^{N}t_{i}^{2}\right)}}$$

## 4. Results

**Figure 6:**
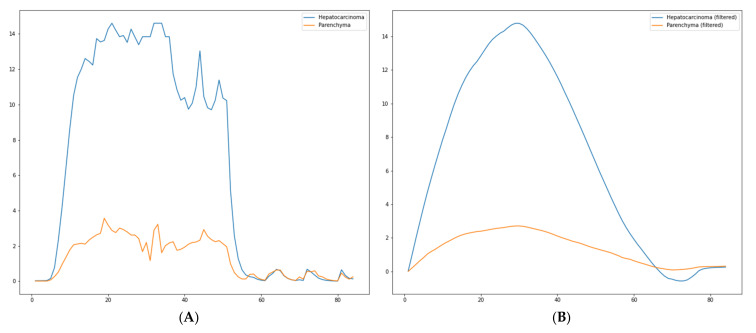
(**A**)—Raw TIC extracted with the proposed method; (**B**)—filtered TIC extracted with the proposed method (patient diagnosed with hepatocarcinoma).

The results presented in this chapter are divided into two parts: the results obtained by extracting the time-intensity curves and the results obtained by the proposed method. For the TIC, several curves are presented for different types of lesions. All the curves are extracted using the proposed method. In Figure 6A, a TIC extracted with the proposed method is presented. The video investigation file was from a patient diagnosed with hepatocarcinoma. Figure 6B shows the same TIC filtered with Savitzky–Golay.

**Figure 7:**
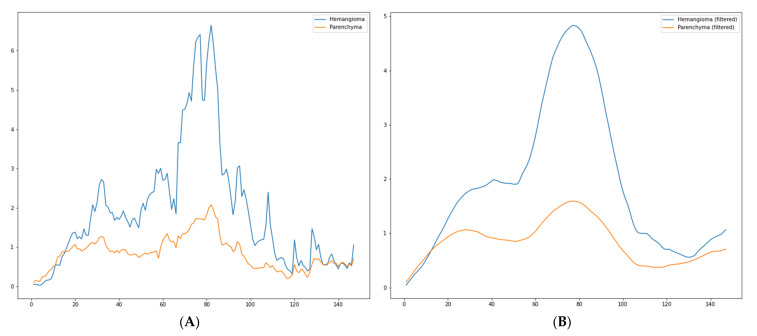
(**A**)—Raw TIC extracted with the proposed method; (**B**)—filtered TIC extracted with the proposed method (patient diagnosed with hemangioma).

In Figure 7A, another TIC extracted with the proposed method is presented. The patient was diagnosed with hemangioma. Figure 7B presents the same TIC but with the filtering applied.

**Figure 8:**
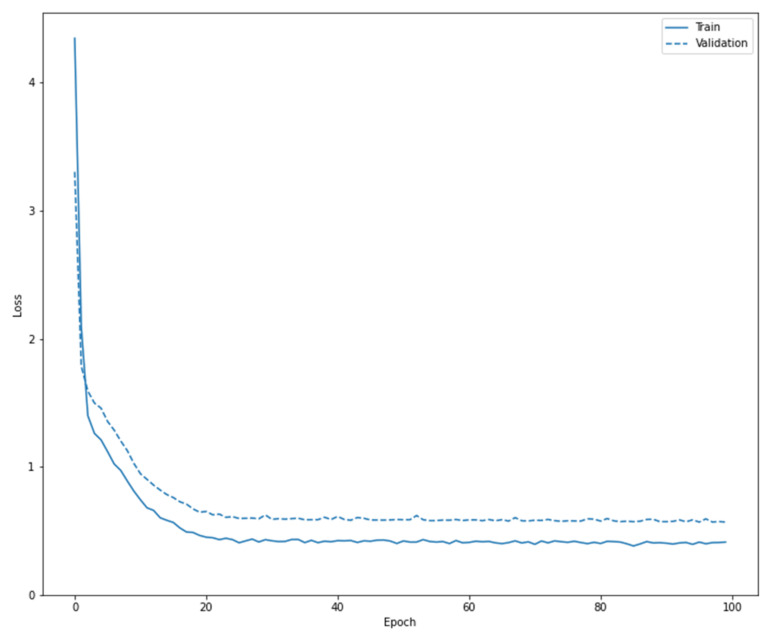
Loss evolution during training and validation.

The results for both training and validation are presented below in Table 6, and the evolution of the loss during training and validation is presented in Figure 8.

## 5. Discussion

The purpose of the presented study is to introduce an automated method of classifying liver lesions based on time-intensity curves and deep learning algorithms in CEUS video investigations. The entire system includes a U-Net segmentation model [11] and a feed-forward neural network model. The segmentation model was trained on B-mode frames extracted from contrast-enhanced ultrasound video investigations. A more detailed description of the model can be found in our previous published study [10]. A feed-forward neural network model was then trained with data from two sources: values extracted from the time-intensity curves and clinical information of the patient. Five output labels were defined for the proposed system: hepatocarcinoma, metastasis, other malignant lesions, hemangioma, and other benign lesions as per liver lesions in our dataset. The ultrasound investigations were performed by the Department of Gastroenterology of the Emergency Clinical County Hospital of Craiova using Hitachi Arietta V 70, convex probe C250. The study involved 49 patients with 59 liver lesions. Most of the patients were male, 31 aged between 38 and 85. The mean tumor size was 51.65 mm, and 22.44% of the patients had a history of previous malignancy. The lesions in the study included hepatic hemangioma, liver cysts, focal nodular hyperplasia, liver adenoma, liver abscess, hepatocellular carcinoma, liver metastases, cholangiocarcinoma, and malignant liver adenoma. The most dominant diagnosis was hepatocellular carcinoma (40.67%), while the least dominant diagnosis was liver adenoma (1.69%). A more detailed description of the patient cohort was presented in our previous studies [10,20]. Being modular, the proposed system can be enhanced to detect other liver lesions by performing transfer learning only on the feed-forward neural network model. In their study, Hang-Tong Hu et al. [21] trained a deep learning model to classify focal liver lesions as malignant or benign. Their cohort contained 363 patients with CEUS video investigations from four phases: plain scan, arterial image, portal image, and delayed image. Residual neural network architecture (ResNet) was used. Their results showed that the trained DL model had a performance comparable with senior radiologists [21]. In other study, Kaizhi Wu et al. [22] introduced a classification system of liver lesions based on CEUS investigations. Their study contained 22 patients with 26 lesions. For extracting the TICs, sparse non-negative matrix factorizations were used, and for classifying the lesions, the TICs were analyzed using a DL model. Their presented results showed that the proposed method outperformed other classifiers such as support vector machine (SVM) or K-nearest neighbors (KNN). B. Schmauch et al. [23] proposed a method for diagnosis of focal liver lesions in ultrasound investigations based on a ResNet50 DL model with an attention block. While their dataset contained only 367 liver images, the results presented showed that the DL model can perform class separation (benign and malignant) with high accuracy. Cătălin Daniel Căleanu et al. [24] proposed a convolutional neural network architecture which achieved high accuracy in classifying liver lesions in CEUS investigations. Compared with other studies, their proposed method could classify an investigation into five different classes (hepatocellular carcinoma, hypervascular metastases, hypovascular metastases, hemangiomas, and focal nodular hyperplasia). Our proposed method contains a TIC extraction component and a feed-forward neural network classifier. Therefore, the presented method analyzes the CEUS video investigation (for extracting the TIC) and the clinical data for each patient. It requires minimal intervention from the human operator as the frames are extracted from each video investigation file. The results presented in Table 6 and Table 7 show that the feed-forward classifier cannot predict with high accuracy the exact type of lesion. However, the model can perform class separation between malignant and benign lesions. Two random samples were selected for analyzing: one from the malignant lesions and one from the benign lesions. The video investigations for each patient were run though the proposed system. The results are presented in Table 7. The first selected patient (PATIENT1) was a female, 79 years old, with a cirrhotic liver. In our dataset, the patient was diagnosed with hepatocarcinoma. The second patient (PATIENT2) was a 62-year-old male with no history of cirrhosis or hepatitis. In our dataset, this patient was diagnosed with hepatic adenoma.

The results presented in Table 6 and Table 7 show that the proposed system is able to perform class separation between malignant and benign. However, in some cases, the system has difficulties detecting a specific lesion in these classes, especially in benign ones. This can be due to relatively low samples of benign lesions in our dataset.

While other studies [22] proposed binary classifiers (benign or malignant lesions), the presented system can classify liver lesions from CEUS video investigations into five classes. Besides CEUS video investigations, it uses clinical data of the patient to perform the classification. Because it uses a DL image segmentation model, it requires no intervention from the human operator during data analysis for prediction. U-Net, the image segmentation model used in the proposed system, was tested in our previous study [10] in terms of execution time and GPU requirements. It can perform an image segmentation in between 32.50 and 76.43 milliseconds (depending on the GPU) [10]. The feed-forward classifier added in the current study has 203 trainable parameters. Thus, together with the TIC extraction module, the group adds a small overhead to the entire system. Analyzing the evolution of the ultrasound signal over time is not new in the medical field. However, generating the graphical representation manually is a difficult task and can introduce errors [25,26]. Automatically extracting TICs from CEUS liver investigations by using either image processing techniques or AI algorithms is an active research area. Simona Turco et al. [27] proposed a method with minimal manual input from the operator. The authors avoided motion compensation by using spatial and spatiotemporal analysis. While the metrics observed in their study showed that it can separate classes with high accuracy, the method could classify the lesion into two classes: malignant or benign. A limitation of our study is the unbalanced classes. Separating the labels from the dataset from binary (malignant or benign) to multiclass (five classes—three malignant and two benign) unbalanced the dataset even more. To counter this, we used the focal cross-entropy loss function, which is usually used in highly unbalanced classes. In addition, we applied oversampling techniques to the dataset.

## 6. Conclusions

The aim of the study was to develop an automated method for classifying liver lesions in CEUS video investigations. A dataset provided by the University of Medicine and Pharmacy Craiova with 50 CEUS video investigations was used. The proposed method included three components or modules: a lesion segmentation module, a TIC extraction module, and a classifier module. A U-Net segmentation model was trained to perform lesion segmentation in B-mode frame-by-frame from the video investigation. By using this segmentation model, tracking the lesion’s movement in the CEUS investigation was not an issue because the segmentation model predicted the mask frame-by-frame. Because we knew the coordinates of each window type in the video frame, B-mode or contrast mode images could be extracted from it. Dilation was applied on each analyzed frame, and together with the predicted mask from the segmentation model, a TIC could be extracted. A feed-forward neural network model was trained with the parameters extracted from the TICs and with clinical data from each patient in the study. The results show that due to class imbalance, the classifier was not able to predict with high accuracy a specific lesion from the minority classes. However, in the majority of classes, the classifier could predict the lesion type with high accuracy. The proposed system can be a useful tool for gastroenterologists or medical students to either provide a prediction of the lesion type or to automatically extract TICs.

## Figures and Tables

**Table 1:** Demographic information of the patients.

	Male	Female	Whole Lot
	31	18	49
Median Age	67	65	67
Minimum Age	38	39	38
Maximum Age	85	83	85
Underlying liver disease			
Liver cirrhosis	24 (77.42%)	15 (83.34%)	39 (79.6%)
Liver hepatitis	7 (22.58%)	3 (16.66%)	10 (20.4%)
Number of tumors			
Single tumor	17 (54.84%)	8 (44.45%)	25 (51.02%)
Multiple tumors	14 (45.16%)	10 (45.55%)	24 (48.98%)

**Table 2:** Coordinates determined experimentally for B-mode and contrast mode.

	X Min	X Max	Y Min	Y Max
B Mode	0	400	78	525
Contrast Mode	400	800	78	525

**Table 3:** Coordinates determined experimentally for the color scale.

	X Min	X Max	Y Min	Y Max
Color scale	770	775	100	170

**Table 4:** Dataset fields used for training the feed-forward neural network model.

Field Name	Type	Comments
Gender	Numerical	Female—encoded as 2 Male—encoded as 1
Age	Numerical	
Cirrhosis History	Boolean	
Hepatitis History	Boolean	
Maximum Intensity	Numerical	
Area Under the Curve	Numerical	
Mean Transit Time	Numerical	
Time to Peak	Numerical	

**Table 5:** Dataset labels used for training the feed-forward neural network model.

Label Name	Type
Hepatocarcinoma	Boolean
Metastasis	Boolean
Other malignant lesions	Boolean
Hemangioma	Boolean
Other benign lesions	Boolean

**Table 6:** Performance results for the feed-forward neural network.

	Categorical Accuracy	F1 Micro	F1 Macro	MCC
Training	0.6287	0.6287	0.5149	0.5744
Validation	0.5863	0.5765	0.5263	0.5243

**Table 7:** Results for two samples selected from the study.

	PATIENT1 (Malignant)	PATIENT2 (Benign)
Hepatocarcinoma	0.78	0.01
Metastasis	0.02	0.007
Other malignant lesions	0.17	0.1
Hemangioma	0.006	0.42
Other benign lesions	0.002	0.46

## Data Availability

The data presented in this study are available on request from the corresponding author. The images were made available from the University’s repository as an anonymized dataset.
